# Simulated Microgravity Alters Gene Regulation Linked to Immunity and Cardiovascular Disease

**DOI:** 10.3390/genes15080975

**Published:** 2024-07-24

**Authors:** Candice G. T. Tahimic, Sonette Steczina, Aimy Sebastian, Nicholas R. Hum, Metadel Abegaz, Masahiro Terada, Maria Cimini, David A. Goukassian, Ann-Sofie Schreurs, Tana M. Hoban-Higgins, Charles A. Fuller, Gabriela G. Loots, Ruth K. Globus, Yasaman Shirazi-Fard

**Affiliations:** 1 Department of Biology, University of North Florida, Jacksonville, FL 32224, USA; 2 Space Biosciences Division, NASA Ames Research Center, Moffett Field, CA 94035, USAmetadel.f.abegaz@gmail.com (M.A.); yasaman.shirazi-fard@nasa.gov (Y.S.); 3 Blue Marble Space Institute of Science, Seattle, WA 98104, USA; 4 Lawrence Livermore National Laboratory, Livermore, CA 94550, USAgloots@ucdavis.edu (G.G.L.); 5 Universities Space Research Association, Washington, DC 20024, USA; 6 Temple University School of Medicine, Philadelphia, PA 19140, USA; maria.cimini@temple.edu; 7 Cardiovascular Research Institute, Icahn School of Medicine at Mount Sinai, New York, NY 10029, USA; david.goukassian@mssm.edu; 8 Department of Neurobiology, Physiology and Behavior, University of California Davis, Davis, CA 95616, USA; 9 Department of Orthopedic Surgery, University of California Davis Health, Sacramento, CA 95817, USA

**Keywords:** hindlimb unloading, microgravity, cardiovascular system, immune response, transcriptomics

## Abstract

Microgravity exposure induces a cephalad fluid shift and an overall reduction in physical activity levels which can lead to cardiovascular deconditioning in the absence of countermeasures. Future spaceflight missions will expose crew to extended periods of microgravity among other stressors, the effects of which on cardiovascular health are not fully known. In this study, we determined cardiac responses to extended microgravity exposure using the rat hindlimb unloading (HU) model. We hypothesized that exposure to prolonged simulated microgravity and subsequent recovery would lead to increased oxidative damage and altered expression of genes involved in the oxidative response. To test this hypothesis, we examined hearts of male (three and nine months of age) and female (3 months of age) Long–Evans rats that underwent HU for various durations up to 90 days and reambulated up to 90 days post-HU. Results indicate sex-dependent changes in oxidative damage marker 8-hydroxydeoxyguanosine (8-OHdG) and antioxidant gene expression in left ventricular tissue. Three-month-old females displayed elevated 8-OHdG levels after 14 days of HU while age-matched males did not. In nine-month-old males, there were no differences in 8-OHdG levels between HU and normally loaded control males at any of the timepoints tested following HU. RNAseq analysis of left ventricular tissue from nine-month-old males after 14 days of HU revealed upregulation of pathways involved in pro-inflammatory signaling, immune cell activation and differential expression of genes associated with cardiovascular disease progression. Taken together, these findings provide a rationale for targeting antioxidant and immune pathways and that sex differences should be taken into account in the development of countermeasures to maintain cardiovascular health in space.

## 1. Introduction

Spaceflight leads to cardiovascular deconditioning in the absence of mitigation strategies. Cardiovascular changes in response to spaceflight are attributed to altered microgravity levels and the ensuing cephalad fluid shift [1]. Overall reductions in physical activity and other factors such as nutritional changes, elevated CO_2_ levels, and a demanding workload also may be contributing factors. Some of the reported cardiovascular changes caused by exposure to the spaceflight environment include reductions in left ventricular mass [2,3], transient atrial distension [4], and hypovolemia [5]. Stagnant or retrograde venous flow as well as thrombosis have been observed in crew during flight [6]. Heart rhythm disturbances postflight also have been reported [7]. Orthostatic intolerance [8] and stiffer carotid arteries [9] have also been observed in crew upon return to Earth. Crew on exploration-class missions (e.g., trip to Mars) will experience prolonged periods of microgravity and exposure to low doses of space radiation given current spacecraft shielding designs. Therefore, it is important to understand the cardiovascular responses to long-duration spaceflight to mitigate potential health risks.

The rodent hindlimb unloading (HU) model [10,11] has been used to gain insights into some aspects of human cardiovascular responses to microgravity and cephalad fluid shifts. HU for four weeks in rats resulted in decreased left ventricular mass and volume [12], consistent with findings from a number of bed rest studies [2,13,14,15,16] and from spaceflight [3]. Two weeks of HU in rats perturbs day–night blood pressure (BP) and heart rate regulation [17]. Compared to baseline (pre-HU) values, HU generally led to increased systolic and diastolic BP during both day and night cycles, whereas heart rate was increased during the day but not at night. Some of these HU findings could also have been confounded by other factors such as social isolation and restraint stress [17]. In addition, arrhythmias and aberrant intracellular Ca^2+^ signaling have been observed in mice that underwent HU (26–56 days) [18]. Changes in redox balance have also been reported. For example, hearts from male Sprague–Dawley rats that underwent two weeks of HU displayed increased levels of malondialdehyde, a lipid peroxidation marker [19].

Currently, there is limited information regarding the effects of long-duration spaceflight on the human cardiovascular system at time scales relevant to interplanetary missions (>2 years). In addition, the effects of age, sex, duration of exposure, and long-term recovery on cardiovascular outcomes are not well established. Hence, the goals of our study are to (1) determine the impact of sex, age, duration of exposure, and recovery on oxidative stress responses to microgravity and (2) define underlying molecular mechanisms. We hypothesized that exposure to simulated microgravity and the ensuing recovery would lead to changes in redox balance and the expression of select genes including those that play a role in oxidative stress responses. To test this hypothesis, we examined hearts from male and female rats that underwent hindlimb unloading (HU) for various durations (up to 90 days) and those that underwent reambulation after 90 days of HU. To achieve this, we assessed DNA oxidative damage markers and select transcripts, and performed RNAseq analysis of cardiac tissue.

## 2. Materials and Methods

### 2.1. Animals and Experiment Design

Animal experiments were carried out in accordance with the recommendations of Guide for the Care and Use of Laboratory Animals, 8th edition. All animal experiments were conducted with prior approval from the UC Davis Institutional Animal Care and Use Committee (IACUC). Female and male Long–Evans rats (Taconic) were selected for this study. Animals were group-housed in standard vivarium cages until the onset of HU. A group of females and males (referred to as “young”) underwent HU at three months of age, while a group of retired male breeders (referred to as “older males”) began HU at nine months of age. Normally loaded (NL) controls were individually housed in standard vivarium cages. Animals were suspended onto the pulley apparatus via orthopedic traction tape as described previously [11]. NL controls were handled the same way as the HU animals except for the actual attachment of traction tape and tail suspension. HU was conducted for either 14 days or 90 days. A subset of nine-month-old (older) males that underwent HU for 90 days were then released from the suspension apparatus to reambulate normally (Reloading, Rel) for 90 days. All animals were supplied with standard rodent laboratory chow (Purina, St. Louis, MO, USA) and water ad libitum. Enrichment consisted of Diamond Twists paper sticks (Harlan, Indianapolis, IN, USA) and I-Chews (Newco, Hayward, CA, USA) and replenished as needed. Room temperature was maintained within a range of 20–26 °C with a 12-h light and 12-h dark cycle. After the designated duration of HU, animals were euthanized via isoflurane overdose followed by exsanguination and decapitation. Tissues were collected shortly thereafter. Hearts were bisected, flash-frozen in liquid nitrogen, and stored at −80 °C until processing. Refer to Table S1 for the sample sizes of the experiment groups included in this study. Young male and female, 90 day HU, and recovery groups were not included in the study either due to insufficient sample sizes or unavailability of appropriately processed samples.

### 2.2. Processing of Frozen Heart Samples

Frozen hearts were retrieved from −80 °C storage and placed on a metal platform that was pre-chilled with liquid nitrogen. Left ventricular tissue was excised from the anterior to posterior ends (apex) of the heart. The excised left ventricular tissue was further cut into thirds (anterior, middle, and posterior). The anterior and posterior thirds were processed for DNA and RNA extraction, respectively. Samples were excluded from analyses if anatomical landmarks could not be conclusively identified for isolation of left ventricular tissue.

### 2.3. RNA Extraction from Cardiac Tissue

Total RNA was extracted from frozen left ventricular tissue using the RNeasy Fibrous Tissue Mini Kit (Qiagen, Germantown, MD, USA) according to manufacturer’s protocol. RNA concentration was determined using NanoDrop 2000 spectrophotometer (Thermo Scientific, Waltham, MA, USA), and purity was determined using the Agilent 2100 Bioanalyzer (Agilent Technologies, Santa Clara, CA, USA). Only samples showing an RNA integrity number (RIN) > 8 were used for RNA sequencing and quantitative polymerase chain reaction (qPCR).

### 2.4. cDNA Synthesis and qPCR

First-strand DNA was synthesized from total RNA as follows. A total of 450 ng of RNA was used for a 30 uL first strand synthesis. Briefly, 1.5 uL of random hexamers (Invitrogen, Waltham, MA, USA, Cat# N8080127), 1.5 μL dNTPs (Qiagen, Cat# 1005631), and 12 μL of water were mixed, incubated at 65 °C for 5 min, and immediately chilled on ice. A solution-containing 3 μL RNase-free water, 6 μL 5× first strand buffer (Invitrogen, Cat# Y02321), 3 μL DTT (Invitrogen, Cat# Y00147), and 1.5 μL RNase Out (Invitrogen, Cat# 100000840) was added to the abovementioned mix and then incubated at 37 °C for 2 min. Then 1.5 μL of Moloney Murine Leukemia Virus (MMLV) reverse transcriptase (200 U/μL, Invitrogen, Waltham, MA, USA, Cat# 28025013) was added to the resulting solution and incubated as follows: 25 °C for 10 min, 37 °C for 50 min, and 70 °C for 15 min. All incubation steps were performed using a TC-312 thermocycler (Techne, Vernon Hills, IL, USA). First-strand DNA was stored at −80 °C until use for qPCR using the Taqman system. Multiplex PCR reactions were set up to measure both genes of interest and housekeeping gene expression in the same well of a 384-well plate. A premade gene expression master mix (Taqman, LifeTechnologies, Waltham, MA, USA, Cat# 4369016) was utilized, and each reaction was completed in duplicate. FAM-labeled Taqman probes (LifeTechnologies, Waltham, MA, USA) were used for the following genes of interest: nuclear factor, erythroid 2-like 2 (*Nfe2l2,* Rn00582415_m1), superoxide dismutase 1 (*Sod1*, Rn00566938_m1), superoxide dismutase 2 (*Sod2*, Rn00690588_g1), and sirtuin 1 (*Sirt1*, Rn01428096_m1). VIC-labeled ribosomal protein L19 (*Rpl19*, Rn00821265_g1) was used as housekeeping gene. qPCR was performed on a QuantStudio 6 Flex Real-Time PCR instrument (Applied Biosystems, Waltham, MA, USA). The relative gene expression levels for HU groups relative to age- and sex-matched controls were determined by the ΔΔCt method and reported as fold change.

### 2.5. 8-Hydroxydeoxyguanosine (8-OHdG) Assay

Total DNA was extracted from frozen left ventricular tissue using the DNeasy Blood and Tissue Kit (Qiagen) according to manufacturer’s protocol. Quantification of 8-OHdG was performed using OxiSelect™ Oxidative DNA Damage ELISA Kit (Cell Biolabs Inc., San Diego, CA, USA, Cat# STA-320) following manufacturer’s specifications. Enzyme reaction/absorbance was measured using a Spectra Max 250 (Molecular Devices, San Jose, CA, USA) and Softmax Pro 5 software.

### 2.6. RNA Sequencing and Bioinformatics Analysis

Poly(A)+-enriched cDNA libraries were prepared using the Illumina TruSeq RNA Library Prep kit (Illumina Inc., Hayward, CA, USA) and sequenced using Illumina NextSeq 550 (Illumina Inc., Hayward, CA, USA) instrument to generate 75 bp single-end reads. Sequencing data quality was checked using FastQC (version 0.11.5) software. Subsequently, reads were mapped to the rat reference genome (rn6) using STAR (version 2.6.0c). Then, a matrix of read counts per gene was generated using “featureCounts” from the Rsubread package (version 1.30.5). Genes with low expression values were filtered from downstream analysis by requiring more than 5 reads in at least 6 samples for each gene. Subsequently, between-sample normalization was performed using EDASeq (version 2.16.0). One sample from each group had to be excluded from analysis due to technical issues. Hence, 6 samples per group were used for the succeeding analysis. RUVseq (version 1.16.0) [RUVs with k = 4] was used to estimate the factors of unwanted variation. Genes differentially expressed between HU and control samples were identified using DESeq2 (version 1.22.2), controlling for factors of unwanted variation. Hierarchical clustering analysis was performed and heat maps generated using Morpheus, with Pearson method selected [20]. RStudio analysis software (Version 1.1.419), together with ggplot2 (version 3.1.1), ggrepel (version 0.8.0), and Bioconductor (DESeq2 version 1.22.2) packages, were used to obtain principal component analysis plots. For gene enrichment analysis, differentially expressed genes meeting a log2 fold change cut-off of >0.3 or <−0.3 and an adjusted *p*-value < 0.05 were input into Toppfun [21,22]. Toppfun was used to assess functional enrichment of DEGs based on ontologies, gene family, phenotype (human disease), and pharmacome (drug–gene associations). Of the 125 differentially expressed genes (DEGs), 121 matched the contents of the Toppfun database and were therefore included in the list for gene enrichment analysis. The DEGs that had no match in the Toppfun database were *Clec4a1*, *Lilrb3l*, *Fcna*, and *Mill1*. Gene enrichment analysis was performed at a false discovery rate (FDR, Benjamini Hochberg) of <0.05 and a gene limit of 2 or greater. GO Terms were plotted for visualization using the matplotlib.pyplot, seaborn, and pandas packages run in Google Colaboratory (Python ver 3.10).

### 2.7. Statistical Analysis

Equivalence of variance was first evaluated by Levene’s test. Once equal variance was confirmed, a *t*-test was performed to compare HU and matched control groups. This study involved an extended sampling period (3 years). To minimize batch effects, we compared HU and the matched controls for any given sex and timepoint since these groups were collected within the same period. Specifically, any HU group and its normally loaded control group (e.g., young male 14 day HU and NL groups) were euthanized on the same day or a day apart. Comparisons were not made across timepoints, sexes, or ages. Statistical analyses were performed using JMP software version 13.1.0 (SAS Institute Inc., Cary, NC, USA). Data shown are mean +/− S.D.

## 3. Results

**Figure 1:**
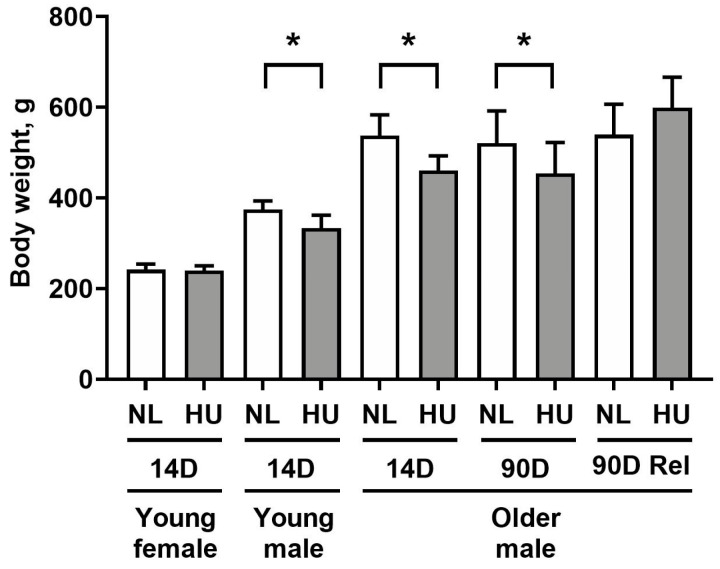
Body weights of animals included in this study. NL: normally loaded; HU: hindlimb unloading; Rel: reambulated (reloading). The HU group was compared to their age- and sex-matched NL controls by Student’s *t*-test. Sample sizes: N = 5–8/group for all groups except for 90D older males, where N = 3. Values depicted are means and standard deviation. * Significant at *p* < 0.05 by Student’s *t*-test.

In this study, we made use of the rodent HU model to gain insight on the effects of microgravity exposure on molecular signatures of the heart. With the exception of the 14-day HU young females, all HU groups had lower body weights versus age- and sex-matched controls (Figure 1), consistent with previous reports [23,24,25]. Weight loss in HU groups of older males was transient, with body weights of NL and HU groups being comparable after 90 days of reambulation. Due to the modest decrease in body weight (10–13%), we do not expect weight loss to be a major confounding factor in other experimental outcomes.

**Figure 2:**
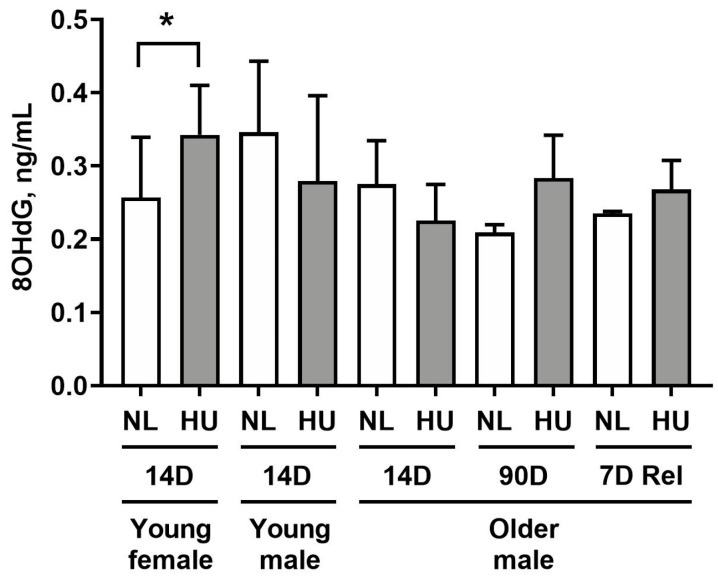
8-hydroxydeoxyguanosine (8-OHdG) levels in left ventricular tissue as measured by ELISA. NL: normally loaded control, HU: hindlimb unloading, 7D Rel: 90D HU + 7D reloading and normally loaded control (NL). The HU groups were compared to their age- and sex-matched NL controls by Student’s *t*-test. Sample sizes: N = 5–8/group for all groups except for 90D older males, where N = 3. Values depicted are means and standard deviation. * Significant at *p* < 0.05 by Student’s *t*-test.

To determine whether simulated microgravity leads to alterations in redox balance in cardiac tissue, we measured levels of 8-hydroxydeoxyguanosine (8-OHdG) in left ventricular tissue (Figure 2). 8-OHdG is a marker for oxidative damage to DNA. Young females after 14 days of HU displayed a modest yet statistically significant increase in 8-OHdG levels (33.2%) compared to age- and sex-matched controls. At 14 days of HU, no differences were observed between HU and control groups in young and older males. Older males at 90 days of HU did not exhibit any significant difference in 8-OHdG levels versus corresponding controls. We further hypothesized that reloading will lead to increased oxidative damage to hearts due to altered fluid distribution and increased functional demand on the heart. Hence, we examined 8-OHdG levels at an earlier timepoint of reambulation (7 days) where we found no differences in 8-OHdG levels in older males that underwent normal reambulation after 90 days of HU relative to controls.

**Figure 3:**
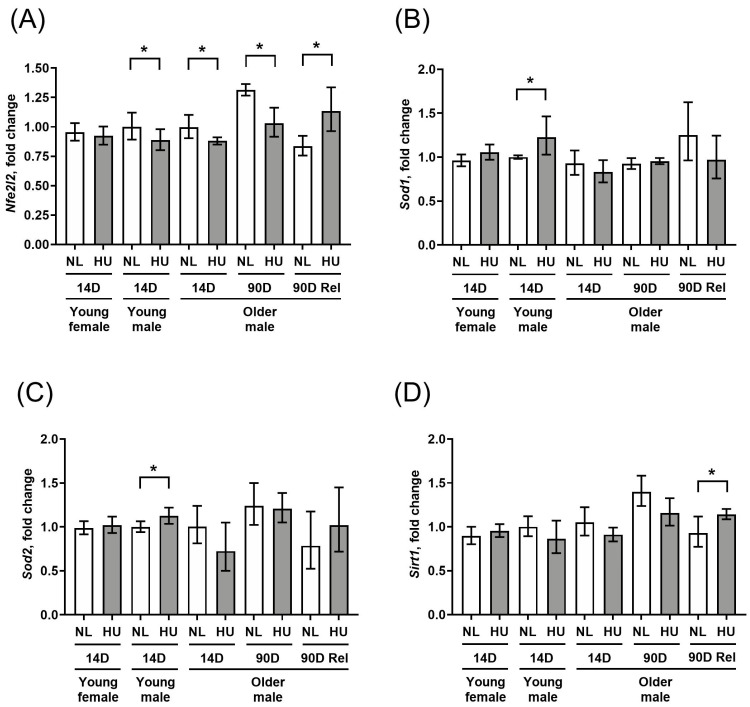
Transcript levels of (**A**) *Nfe2l2*, (**B**) *Sod1*, (**C**) *Sod2*, and (**D**) *Sirt1* in left ventricular wall as measured by qPCR. Values depicted are mean fold changes relative to young male control at 14 days of treatment as determined by the ΔΔCt method. Errors bars show upper and lower ranges. NL: normally loaded control, HU: hindlimb unloading, 90D Rel: 90D HU + 90D reloading and NL control. Sample sizes: N = 3–7/group. * Significant at *p* < 0.05 by Student’s *t*-test by comparing HU with age- and sex-matched NL control.

To further assess any changes in key molecular determinants of redox balance, we measured gene expression levels of select antioxidant genes (*Sod1* and *Sod2*) as well as a master transcriptional regulator of the antioxidant response, *Nfe2l2* (Figure 3A–C). In addition, we examined the gene expression of *Sirt1*, a marker of cellular senescence and oxidative stress also known to impact longevity [26,27] (Figure 3D). Compared to sex- and age-matched controls, young females that underwent 14 days of HU did not show any changes in gene expression for any of the four genes tested. Young males at the same timepoint showed a modest downregulation of *Nfe2l2* (Figure 3A) as well as upregulation of *Sod1* and *Sod2* (Figure 3B and 3C respectively), but no change in *Sirt1* expression (Figure 3D). In older males, *Nfe2l2* was downregulated at 14 days of HU, while there were no changes in *Sod1*, *Sod2,* or *Sirt1* expression. The downregulation of *Nfe2l2* expression in older males persisted at 90 days of HU. After a 90-day reambulation period, this pattern was reversed, with HU groups showing upregulated expression of *Nfe2l2* and *Sirt1* relative to controls.

**Figure 4:**
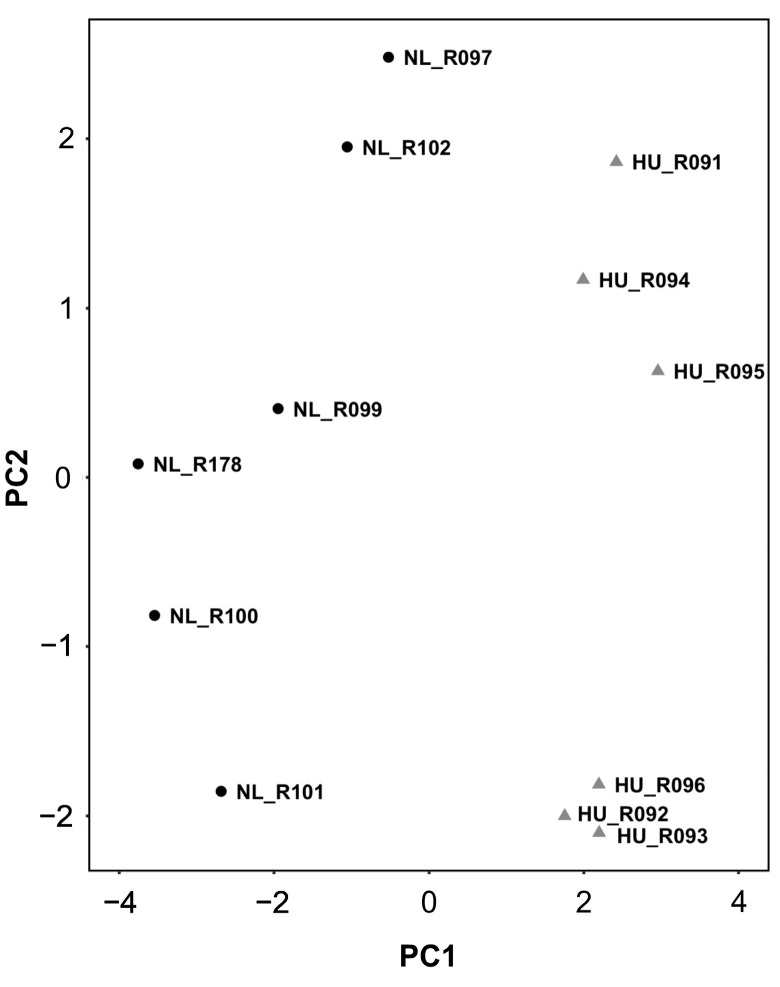
PCA plot of transcriptomic data from older males that underwent 14 days of HU (gray triangles) and corresponding NL controls (black circles). Animal ID is indicated by the letter “R” succeeded by numbers.

**Figure 5:**
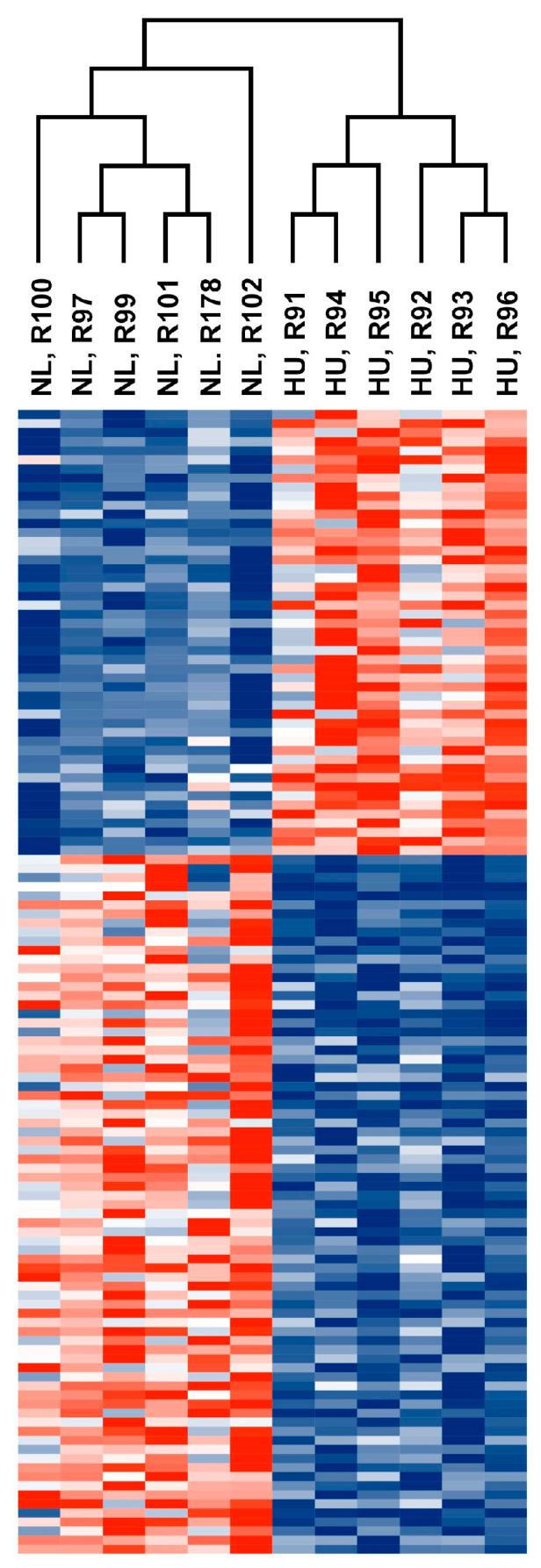
Heatmap showing normalized counts of differentially expressed genes from older male 14D NL and HU groups. Each cell corresponds to a gene. Red: Upregulated in 14D HU relative to NL group. Blue: Downregulated in 14D HU relative to NL group. Magnitude of upregulation or downregulation is proportional to the intensity of red or blue. Deepest red: most upregulated; deepest blue: most downregulated.

**Figure 6:**
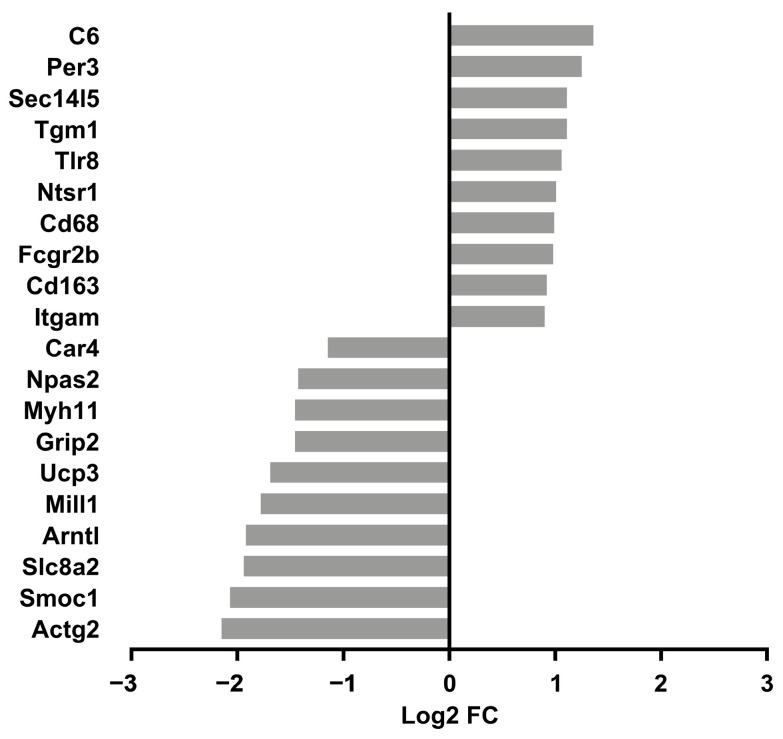
Top 10 upregulated and downregulated genes in older male 14D HU relative to NL groups. Log2 FC: Log2 fold change. Refer to Table 1 for full list of DEGs.

We reasoned that identifying the molecular signature of the early adaptation of cardiac tissue to HU will allow us to link early changes in gene expression and biological pathways to later cardiac outcomes. We therefore conducted transcriptomic analysis (RNAseq) of tissue from the left ventricle in older (nine-month-old) males and their corresponding controls. We selected nine-month-old males for analysis (samples were not available for females of similar age) over the other three-month-old groups. Principal component analysis (PCA) and hierarchical clustering generally indicated clustering by treatment group (Figure 4 and Figure 5 respectively). A total of 125 genes were differentially expressed in the HU group relative to controls. Of these, 48 were upregulated and 77 were downregulated (Table 1). The top 10 upregulated and downregulated genes included a number of immune-related genes such as *Tlr8* and the macrophage/monocyte markers *CD68* and *CD163* as well as two circadian clock-related genes, *Per3* and *Arntl* (Figure 6). Fifteen of these DEG’s were cluster-of-differentiation (CD) molecules belonging to the C-type lectin domain family, while 6 were LY6/PLAUR domain-containing members of the complement gene family (Table 2). *Nfe2l2* did not appear as a downregulated gene in the 14D HU hearts of older males based on RNAseq despite the observed decrease (although modest) in transcript levels by qPCR analysis (Figure 3A, Table 1). This is likely due to differences in the normalization methods for the two assays.

**Figure 7:**
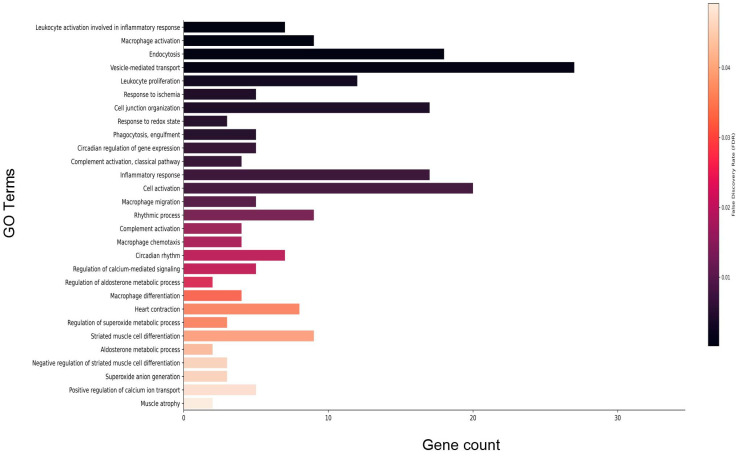
Select enriched gene ontology (GO) terms for biological processes in older male 14D HU group relative to NL group. Gene count refers to the number of DEGs that matched the GO term. The vertical bar represents the color scale of the FDR with black representing the lowest FDR. Refer to Table S2 for the full list of GO terms.

Gene enrichment analysis of DEGs that met the FDR threshold revealed a predominance of immune-related processes including macrophage activation and complement activation. Further, circadian signaling, vesicle-mediated transport, and redox signaling were differentially enriched (Figure 7; refer to Table S2 for full list). Gene enrichment analysis for disease phenotypes using Toppfun [21,22] also revealed enrichment of a subset of DEGs linked to cardiovascular disease (CVD), immune dysfunction, neurovascular disease, cancer, metabolic disease, and sleep disorders. Cardiovascular pathologies represented include hypertensive disease, myocardial infarction, and heart failure. Immune disorders include complement deficiency and the autoimmune disease lupus erythematosus (Table 3; refer to Table S3 for full list). Consistent with the human disease phenotype findings, 24 DEGs were associated with the anti-bradycardia drug isoproterenol, a non-selective β adrenergic receptor agonist. In addition, a number of DEGs were associated with anti-hypertensive drugs losartan and valsartan, both angiotensin receptor blockers. A subset of DEGs also were associated with simvastatin (anticholesterolemic), doxorubicin (cancer treatment), rosiglitazone (antidiabetic), and melatonin (sleeping aid) (Table 4; refer to Table S4 for full list).

## 4. Discussion

The objective of this study was to determine the impact of simulated microgravity on redox signaling in the heart and the overall transcriptomic landscape. Our findings from a rat hindlimb unloading (HU) model for cephalad fluid shift and microgravity exposure suggest sex and age differences in oxidative stress responses. Young males displayed upregulation in the expression of the antioxidants *Sod1* and *Sod2*, and a decrease in *Nfe2l2* expression, with no changes in 8-OHdG levels. On the other hand, increased levels of the DNA oxidative marker 8-OHdG was observed in females that underwent 14 days of HU with no changes in the expression levels of the redox signaling-related genes examined. One interpretation of our findings is that during short-term HU, young males mitigate oxidative damage more effectively than young females. Alternatively, it is possible that the kinetics of the emergence and repair of oxidative damage are sexually dimorphic. It is also unclear whether sex differences in oxidative stress responses persist due to unavailability of samples at later timepoints. Further, short-term HU in older males does not appear to enhance oxidative damage. Although not statistically significant, there was a trend toward increased 8-OHdG in the older males that underwent 90 days of HU compared to controls. We cannot rule out the possibility that long-duration HU can increase oxidative damage in the hearts of older males due to the small sample size available to perform the 8-OHdG assay in these groups. At 14 days of HU, older males exhibited a downregulation of *Nfe2l2* expression, while 8-OHdG levels were not different between HU and controls. The persistent downregulation of *Nfe2l2* at this timepoint is consistent with the observed trend towards increased oxidative damage during prolonged HU. A 90 day reambulation period after prolonged HU of 90 days led to upregulated *Nfe2l2* and *Sirt1* expression. It is possible that upregulation of *Nfe2l2* and *Sirt1* may be one of the underlying mechanisms by which the heart can recover from extended durations of HU. We recognize the extended time period for the conduct of the HU experiments and varying sample sizes across groups as limitations of our study. Specifically, the interpretation of the results from the 8-OHdG and qPCR assays which were performed on multiple HU groups and their corresponding NL controls merits further study. Consistent with our findings, another group has reported that HU elicits an antioxidant response. In the hearts of 6-month-old mice, HU led to time-dependent increases in the oxidation of glutathione to glutathione disulfide, an indicator of oxidative damage [28]. Taken together, our findings and those of others support the use of antioxidant-based countermeasures for extended duration spaceflight.

RNAseq results indicate that 14 days of HU in older males leads to enrichment of processes involved in the immune response, including complement activation and inflammation (Figure 7, Table S2). Complement activation, a component of innate immunity, is a mechanism by which the host defends against microbial infections. Studies in the last decade have revealed a role for the complement system in recognizing damaged host cells and coordinating with other elements of the immune response to achieve resolution to injury [29,30,31]. Dysregulated complement responses have been linked to autoimmune disease [32] as well as poor outcomes in patients with cardiovascular disease [33,34,35] and related animal models [36,37]. Our results indicate upregulation of several complement genes. These include *C1qa*, *C1qb*, and *C1qc*, all components of the membrane attack complex, as well as *C5ar1*, the receptor for the complement anaphylatoxin 5a, which can stimulate immune cells in myocardial infarct models [29,36,37] (Table 1). The implication of upregulated complement response in cardiac tissue during simulated microgravity requires further study given the emerging role of the complement pathway in the development of cardiovascular disease [37,38]. Interestingly, in International Space Station (ISS) crew, elements of the complement response were found to be modestly increased within a week of return to Earth relative to pre-flight values [39]. However, the impact of actual flight on complement responses could not be evaluated, since no in-flight measures were reported in the said study.

The underlying bases for the observed upregulation of complement genes in HU hearts is unclear. In Earth-based cardiovascular disease, altered fluid distribution and pressure changes can give rise to injury of cardiac tissue and therefore may play a role in the upregulated complement response seen in this rodent model for microgravity-induced fluid shifts. Complement activation also can occur as a response to pathogens or their constituents. Hence, another possibility that requires further study is the role of gut permeability in promoting a complement response during simulated microgravity. HU has been reported to alter the gut microbiome, cause damage to intestinal villi, and compromise the structural integrity of tight junctions [40], which can lead to gut leakiness. Indeed, HU can cause a transient release of lipopolysaccharide (LPS) in circulation and changes in the distribution of neutrophils and lymphocytes, [41] as well as an increase in circulating levels of the potent pro-inflammatory cytokine interferon γ (*IFNγ*) [42]. Further, synthetic bacterial DNA has been shown to induce a robust inflammatory response in the heart of mice [43]. Consistent with a potential role of gut permeability, we found upregulation of *CD14* in 14-day HU hearts. *CD14* is a surface receptor expressed in circulating monocytes/macrophages, which binds to LPS to induce an immune response [44]. Other key genes involved in the inflammatory response were also upregulated after 14 days of HU, including *CD163* and *CD68*. *CD163* is a marker for activated macrophages. *CD163*+ macrophages promote angiogenesis, vessel permeability, and leukocyte infiltration [45] and are increased in hearts of simian immunodeficiency virus-infected monkeys [46]. Further, *CD68* marks the monocyte/macrophage population. Both expression levels of *CD68* and immunopositive cell counts increase in the hearts of mice exposed to spaceflight-relevant doses (<0.5 Gy) of radiation [47]. In addition, HU in mice increased hippocampal *CD68* levels, which mark activated microglial populations [48,49]. Collectively, the upregulation of *CD14*, *CD68*, and *CD163* expression would be consistent with macrophage infiltration in HU hearts. Humans in future deep space missions will experience extended periods of low-dose space radiation and microgravity. Hence, the impact of the predicted upregulation of macrophage activity on human cardiovascular health requires further study. *Tlr8* also was upregulated in the hearts of 14-day HU animals. Accumulating evidence demonstrates the important role of TLR signaling in the development of cardiovascular disease [50,51], whereas *Tlr8* transcripts increased in patients with enterovirus-induced dilated cardiomyopathy [52].

RNAseq results also suggest that HU in older males can perturb cardiac circadian clock signals. The circadian clock gene, *Arntl (Bmal1)*, was the fourth most downregulated gene in the HU group, while *Per3* was the most upregulated gene. Other circadian clock-related genes such as *Per2* and *Clock* were upregulated and downregulated, respectively. There is a growing appreciation for the role of clock genes in cardiovascular disease. Cardiac-specific deletion of *Arntl* leads to congestive heart failure during aging, upregulation of oxidative stress-responsive genes in hearts and altered energy metabolism [53]. In addition, *Per2* gene ablation worsens the inflammatory response to myocardial ischemia [54]. ISS crew experience circadian misalignment which leads to sleep–wake cycle disruption [55]. Bioinformatic analysis of tissues from rodent spaceflight and ground-based studies (e.g., skeletal muscle, adrenals, kidney, liver) consistently show that spaceflight and its analogs lead to altered expression of circadian clock-related genes including *Per2*, *Arntl*, and *Clock* [56]. Taken together, our findings and those of others show the importance of exploring the role of circadian clock signaling in spaceflight-induced tissue deficits.

RNAseq also revealed the enrichment of processes related to redox signaling, such as superoxide anion generation. Furthermore, 14 days of HU led to differential expression of a number of genes known to play a role in the defense against oxidative damage. In the current study, Clusterin *(Clu),* also known as Apolipoprotein J (*ApoJ*), was upregulated after 14 days of HU. Overexpression of *Clu* in rat ventricular cells has been reported to rescue angiotensin II-induced ROS production and apoptosis [57]. *Ucp3*, a member of the family of mitochondrial uncoupling proteins (UCPs), was downregulated at 14 days of HU. UCPs have a number of functions, such as dissipating the proton gradient in the mitochondria, which reduces ATP production, releasing energy as heat, and also play a role in the regulation of redox balance [58]. Compared to wild-type controls, the hearts of *Ucp3* knockout mice maintained in thermoneutral conditions have reduced mitochondrial complex activities as well as increased levels of ROS and oxidative stress markers [59]. Thus, the downregulation of *Ucp3* in the hearts of HU rats suggests that simulated microgravity alters redox signaling and energetic processes in the mitochondria, consistent with findings from spaceflight and analog studies of other tissues [60,61]. Regulator of Calcineurin 1 (*Rcan1*), which is downregulated in hearts after 14 days of HU, plays a role in NFAT/Calcineurin signaling and is implicated in CVD progression [62,63]. Deletion of *Rcan1* exacerbates the effects of septic cardiomyopathy and increases cardiac mitochondrial injury, as shown by elevated ROS levels and loss of mitochondrial membrane potential [62]. Taken together, our findings are in support of the hypothesis that simulated microgravity leads to altered redox signaling and function in the heart. In addition, these results are consistent with our earlier findings that hearts of mice that have been in space for 15 days show altered expression of genes regulating redox balance [64]. Taken together, the overall transcriptomic signature in the left ventricle of older males at 14 days of HU (~1 human year) suggests activation of the immune system and pro-inflammatory signaling, altered expression of circadian clock genes, and induction of signals that promote ROS production together with upregulation of select cellular defenses to oxidative damage. Therefore, our model predicts that in the absence of any mitigation strategies, extended spaceflight (>a year) may upregulate pro-inflammatory signaling in cardiac tissue. Consistent with our hypothesis, simulated microgravity led to enhanced expression of genes involved in the inflammatory and oxidative stress responses. Therefore, mitigating excessive inflammation and ensuring optimum antioxidant defenses (e.g., via dietary supplementation) may be a useful approach to ensure long-term cardiac health in astronauts during deep space missions. The upregulation of complement activation genes in hearts of rodents exposed to HU needs further study to determine its underlying basis and long-term consequences for cardiac health. In addition, we have identified the molecular signature of cardiac tissue responses to HU. A subset of these differentially expressed genes could be tested as candidate circulating biomarkers for monitoring the trajectory of an astronaut’s response to spaceflight or predicting the need for medical intervention.

## Figures and Tables

**Table 1:** Differentially expressed genes (DEGs) in older male 14D HU group relative to age- and sex- matched NL control group. DEGs were selected using an adj *p*-value < 0.05 at a log2 fold change cut-off > 0.3 or <−0.3. Total DEGs: 125 (48 upregulated and 77 downregulated).

DEGs	Log2 FC	*p* adj	Gene Name
*Actg2*	−2.15	0.0241	Actin, γ 2, smooth muscle, enteric
*Adhfe1*	−0.53	0.0003	Alcohol dehydrogenase, iron-containing, 1
*Alox5ap*	0.58	0.0454	Arachidonate 5-lipoxygenase activating protein
*Aplnr*	−0.41	0.0457	Apelin receptor
*Aqp7*	−0.77	0.0007	Aquaporin 7
*Arl11*	0.79	0.0357	ADP-ribosylation factor-like GTPase 11
*Arntl*	−1.92	1.77 × 10^−12^	Aryl hydrocarbon receptor nuclear translocator-like
*Asb2*	−0.32	0.0002	Ankyrin repeat and SOCS box-containing 2
*Atp2a1*	−0.77	0.0284	ATPase sarcoplasmic/endoplasmic reticulum Ca2+ transporting 1
*Atp4a*	−1.69	0.0075	ATPase H+/K+ transporting subunit α
*Bcat2*	−0.40	0.0038	Branched chain amino acid transaminase 2
*Bckdha*	−0.40	0.0083	Branched chain ketoacid dehydrogenase E1, α polypeptide
*Bcl6b*	−0.61	0.0055	BCL6B, transcription repressor
*C1qa*	0.57	0.0483	Complement C1q A chain
*C1qb*	0.74	0.0005	Complement C1q B chain
*C1qc*	0.69	0.0007	Complement C1q C chain
*C5ar1*	0.61	0.0483	Complement C5a receptor 1
*Car4*	−1.15	0.0017	Carbonic anhydrase 4
*Cd14*	0.67	0.0314	CD14 molecule
*Cd163*	0.92	0.0004	CD163 molecule
*Cd44*	0.33	0.0353	CD44 molecule
*Cd68*	0.99	0.0059	CD68 molecule
*Cd93*	−0.42	0.0065	CD93 molecule
*Cdkn1a*	−0.75	0.0011	Cyclin-dependent kinase inhibitor 1A
*Cited4*	−0.52	0.0296	Cbp/p300-interacting transactivator, with Glu/Asp-rich carboxy-terminal domain, 4
*Clec4a1*	0.86	0.0121	C-type lectin domain family 4, member A1
*Clock*	−0.44	0.0002	Clock circadian regulator
*Clu*	0.44	0.0055	Clusterin
*Col5a3*	−0.66	0.0280	Collagen type V α 3 chain
*Crispld2*	−0.45	0.0234	Cysteine-rich secretory protein LCCL domain-containing 2
*Csf1r*	0.51	0.0111	Colony stimulating factor 1 receptor
*Dkk3*	−0.30	0.0351	Dickkopf WNT signaling pathway inhibitor 3
*Dot1l*	−0.44	0.0190	DOT1-like histone lysine methyltransferase
*Ecrg4*	−1.27	0.0299	ECRG4 augurin precursor
*Eepd1*	−0.44	0.0007	Endonuclease/exonuclease/phosphatase family domain-containing 1
*Ephx1*	0.84	0.0007	Epoxide hydrolase 1
*Exoc4*	0.35	0.0073	Exocyst complex component 4
*F5*	0.86	0.0345	Coagulation factor V
*Fabp3*	−0.41	1.03 × 10^−5^	Fatty acid-binding protein 3
*Fam160a1*	−0.51	0.0369	Family with sequence similarity 160, member A1
*Fam220a*	−0.40	0.0010	Family with sequence similarity 220, member A
*Fam26e*	−0.67	0.0235	Calcium homeostasis modulator family member 5
*Fcgr2b*	0.98	0.0314	Fc fragment of IgG receptor IIb
*Fcna*	0.63	0.0015	Ficolin A
*Fhl2*	−0.51	0.0121	Four and a half LIM domains 2
*Gnb3*	−0.47	0.0093	G protein subunit β 3
*Gng2*	0.47	0.0457	G protein subunit γ 2
*Gpsm1*	−0.42	0.0063	G-protein signaling modulator 1
*Grip2*	−1.46	0.0022	Glutamate receptor interacting protein 2
*Gsn*	−0.49	0.0008	Gelsolin
*Gstz1*	−0.69	0.0006	Glutathione S-transferase zeta 1
*Hadh*	−0.37	1.60 × 10^−5^	Hydroxyacyl-CoA dehydrogenase
*Heyl*	−0.31	0.0105	Hairy/enhancer-of-split related with YRPW motif-like
*Hspa5*	−0.34	0.0456	Heat shock protein family A member 5
*Inha*	0.71	0.0345	Inhibin subunit α
*Ispd*	−0.44	0.0190	Isoprenoid synthase domain-containing
*Itga6*	−0.36	0.0011	Integrin subunit α 6
*Itgam*	0.90	0.0061	Integrin subunit α M
*Itgb2*	0.80	0.0108	Integrin subunit β 2
*Kank1*	−0.39	0.0307	KN motif and ankyrin repeat domains 1
*Kcnma1*	−2.16	0.0008	Potassium large conductance calcium-activated channel, subfamily M, α member 1
*Laptm5*	0.50	0.0117	Lysosomal protein transmembrane 5
*Lbh*	−0.38	0.0226	Limb bud and heart development
*Lcp1*	0.42	0.0162	Lymphocyte cytosolic protein 1
*Leo1*	−0.34	0.0295	LEO1 homolog, Paf1/RNA polymerase II complex component
*Lgals3*	0.81	0.0077	Galectin 3
*Lilrb3l*	0.82	0.0435	Leukocyte immunoglobulin-like receptor subfamily B member 3-like
*Limd1*	−0.48	0.0015	LIM domain-containing 1
*Lingo4*	−0.55	0.0142	Leucine-rich repeat and Ig domain-containing 4
*Map3k7cl*	−0.66	0.0369	MAP3K7 C-terminal-like
*Mill1*	−1.78	0.0007	MHC I-like leukocyte 1
*Mrc1*	0.70	0.0105	Mannose receptor, C type 1
*Mtfp1*	−0.33	0.0250	Mitochondrial fission process 1
*Mtus1*	−0.30	0.0077	Mitochondrial tumor suppressor 1
*Mx2*	−0.45	0.0004	MX dynamin-like GTPase 2
*Myh11*	−1.46	0.0483	Myosin heavy chain 11
*Myo5b*	−0.46	0.0261	Myosin Vb
*Myom2*	−0.85	1.53 × 10^−14^	Myomesin 2
*Nckap1l*	0.72	0.0091	NCK associated protein 1-like
*Npas2*	−1.43	1.51 × 10^−6^	Neuronal PAS domain protein 2
*Nrp2*	−0.31	0.0457	Neuropilin 2
*Ntsr1*	1.01	0.0405	Neurotensin receptor 1
*Nudt4*	0.37	0.0073	Nudix hydrolase 4
*P2rx4*	0.42	0.0483	Purinergic receptor P2X 4
*Paqr6*	−0.57	0.0250	Progestin and adipoQ receptor family member 6
*Per2*	0.84	1.29 × 10^−9^	Period circadian regulator 2
*Per3*	1.25	0.0226	Period circadian regulator 3
*Phlda1*	−0.65	0.0420	Pleckstrin homology-like domain, family A, member 1
*Pi16*	−0.58	0.0215	Peptidase inhibitor 16
*Pik3ip1*	0.43	0.0043	Phosphoinositide-3-kinase interacting protein 1
*Ppp1r14c*	−0.34	0.0405	Protein phosphatase 1, regulatory (inhibitor) subunit 14c
*Ppp1r3c*	−0.41	0.0065	Protein phosphatase 1, regulatory subunit 3C
*Rapgef5*	0.31	0.0451	Rap guanine nucleotide exchange factor (GEF) 5
*Rasd2*	−0.77	0.0091	RASD family, member 2
*Rasl11b*	−0.59	0.0105	RAS-like family 11 member B
*Rcan1*	−0.63	0.0005	Regulator of calcineurin 1
*Rhobtb1*	0.82	2.92 × 10^−9^	Rho-related BTB domain-containing 1
*Rhoj*	−0.41	0.0210	Ras homolog family member J
*Rimbp2*	−0.62	0.0025	RIMS-binding protein 2
*Rpl3*	0.46	0.0120	Ribosomal protein L3
*Rpl3l*	−0.62	0.0004	Ribosomal protein L3-like
*S100a10*	0.37	0.0154	S100 calcium-binding protein A10
*S100a4*	0.84	1.60 5	S100 calcium-binding protein A4
*Slc38a2*	−0.31	0.0015	Solute carrier family 38, member 2
*Slc41a3*	−0.87	5.13 × 10^−7^	Solute carrier family 41, member 3
*Slc8a2*	−1.94	0.0454	Solute carrier family 8 member A2
*Smyd2*	−0.38	0.0483	SET and MYND domain-containing 2
*Sparcl1*	−0.40	0.0015	SPARC-like 1
*Srebf1*	−0.32	0.0166	Sterol regulatory element-binding transcription factor 1
*Stxbp1*	0.34	0.0038	Syntaxin-binding protein 1
*Tef*	0.74	0.0002	TEF, PAR bZIP transcription factor
*Tf*	0.64	0.0295	Transferrin
*Tfrc*	−0.51	0.0142	Transferrin receptor
*Tgm1*	1.11	0.0084	Transglutaminase 1
*Tlr8*	1.06	0.0053	Toll-like receptor 8
*Tmem176b*	0.31	0.0197	Transmembrane protein 176B
*Tmem179*	−0.51	0.0091	Transmembrane protein 179
*Tp53i11*	−0.42	0.0051	Tumor protein p53 inducible protein 11
*Tpsb2*	−0.78	0.0339	Tryptase β 2
*Trim16*	−0.36	0.0357	Tripartite motif-containing 16
*Tspan18*	−0.57	0.0084	Tetraspanin 18
*Tut1*	0.51	0.0277	Terminal uridylyl transferase 1, U6 snRNA-specific
*Tyrobp*	0.50	0.0365	Transmembrane immune signaling adaptor Tyrobp
*Ucp3*	−1.69	4.13 × 10^−5^	Uncoupling protein 3
*Wee1*	0.47	0.0357	WEE1 G2 checkpoint kinase

**Table 2:** Top gene families represented within DEGs.

Name	FDR	No. of DEGs	Gene Symbol
CD molecules, C-type lectin domain family	1.99 × 10^−8^	15	*Mrc1*, *Pi16*, *Itga6*, *Itgam*, *Itgb2*, *Tlr8*, *Cd163*, *Tfrc*, *Cd93*, *Csf1r*, *Cd14*, *Fcgr2b*, *Cd44*, *Cd68*, *C5ar1*
CD molecules, complement system, LY6/PLAUR domain-containing	4.69 × 10^−7^	6	*Itgam*, *Itgb2*, *C1qa*, *C1qb*, *C1qc*, *C5ar1*
Scavenger receptors	0.0002	4	*Mrc1*, *Cd163*, *Cd14*, *Cd68*
Basic helix–loop–helix proteins	0.0026	5	*Srebf1*, *Clock*, *Heyl*, *Bmal1*, *Npas2*
CD molecules, protein phosphatase 1 regulatory subunits, integrin α subunits	0.0407	2	*Itga6*, *Itgam*
Rho family GTPases	0.0407	2	*Rhoj*, *Rhobtb1*
S100 calcium-binding proteins, EF-hand domain-containing	0.0407	2	*S100a4*, *S100a10*

**Table 3:** Select human diseases associated with a subset of DEGs in this study. The last column indicates the number of DEGs that overlap with the known set of genes associated with human disease. Refer to Table S3 for full list. CVD: Cardiovascular disease.

Classification	Disease	FDR	No. of DEGs
CVD	Libman–Sacks disease (nonbacterial thrombotic endocarditis)	0.0059	4
CVD	Coronary restenosis (is_implicated_in)	0.0150	2
CVD	Abdominal aortic aneurysm (is_marker_for)	0.0208	2
CVD	Myocardial infarction (is_implicated_in)	0.0208	4
CVD	Posterior choroidal artery infarction	0.0218	2
CVD	Peripheral arterial disease	0.0330	3
CVD	Hypertension (is_implicated_in)	0.0336	4
Immune disorder	C1q deficiency	4.50 × 10^−5^	3
Immune disorder	Lupus erythematosus, systemic	0.0013	5
Immune disorder	Complement deficiency disease	0.0125	3
Immune disorder	Systemic lupus erythematosus (implicated_via_orthology)	0.0200	3
Metabolic disease	Obesity	2.83 × 10^−9^	13
Metabolic disease	Endogenous hyperinsulinism	0.0007	4
Metabolic disease	Exogenous hyperinsulinism	0.0007	4
Metabolic disease	Compensatory hyperinsulinemia	0.0007	4
Metabolic disease	Insulin resistance	0.0007	5
Metabolic disease	Insulin sensitivity	0.0007	5
Metabolic disease	Hyperinsulinism	0.0007	4
Metabolic disease	Metabolic syndrome	0.0330	5
Neurovascular disease	Stroke, ischemic	0.0078	2
Neurovascular disease	Ischemic stroke	0.0078	2
Neurovascular disease	Brain ischemia (biomarker_via_orthology)	0.0218	4
Neurovascular disease	Cerebral infarction, left hemisphere	0.0218	2
Neurovascular disease	Anterior choroidal artery infarction	0.0218	2
Neurovascular disease	Cerebral infarction, right hemisphere	0.0218	2
Neurovascular disease	Subcortical infarction	0.0218	2
Neurovascular disease	Cerebral infarction	0.0218	2
Cancer, hematological	Myeloid leukemia, chronic	0.0008	4
Cancer, hematological	Acute myeloid leukemia, m1	0.0014	6
Cancer, hematological	Acute myeloid leukemia (aml-m2)	0.0014	6
Cancer, hematological	Leukemia, myelocytic, acute	0.0057	6
Cancer, hematological	Adult t-cell leukemia/lymphoma (is_marker_for)	0.0181	2
Cancer, hematological	Acute promyelocytic leukemia	0.0218	3
Cancer, hematological	Hematologic neoplasms	0.0258	2
Sleeping disorder	Seasonal affective disorder	0.0002	4
Sleeping disorder	Advanced sleep phase syndrome, familial	0.0042	2
Sleeping disorder	Advanced sleep phase syndrome (is_implicated_in)	0.0059	2

**Table 4:** Select pharmacologicals associated with a subset of DEGs in this study. The list contains current and discontinued drugs. Refer to Table S4 for the full list of pharmacologicals and associated DEGs. CVD: cardiovascular disease, HIV: human immunodeficiency virus, MS: multiple sclerosis.

Drug	Use	No. of DEGs	FDR
Atorvastatin calcium	Anti-CVD, antihypercholesterolemic	7	0.0069
Cerivastatin	Anti-CVD, antihypercholesterolemic	3	0.0371
Simvastatin	Anti-CVD, antihypercholesterolemic	14	0.0007
Abciximab	Antithrombotic	4	0.0002
Losartan	Antihypertensive, angiotensin receptor blocker	6	0.0190
Valsartan	Antihypertensive, angiotensin receptor blocker	6	0.0051
Hydrochlorothiazide	Antihypertensive, diuretic	2	0.0305
Isoproterenol	Anti-bradycardia, non-selective β-adrenergic receptor agonist	24	1.043 × 10^−6^
Muraglitazar	Antidiabetic	11	0.0037
Rosiglitazone	Antidiabetic	27	3.669 × 10^−5^
Tesaglitazar	Antidiabetic	12	0.0025
Troglitazone	Antidiabetic	17	0.0305
Basiliximab	Immune suppressor, organ transplant	4	0.0002
Muromonab	Immune suppressor, organ transplant	4	0.0003
Efalizumab	Anti-auto immune disease, anti-psoriasis	4	0.0001
Etanercept	Anti-auto immune disease, anti-psoriasis	4	0.0002
Alefacept	Anti-auto immune disease, psoriasis	4	0.0001
Rituximab	Anti-Rheumatoid arthritis	4	0.0001
Adalimumab	Anti-rheumatoid arthritis, anti-Crohn’s disease, anti-psoriasis	4	0.0001
Palivizumab	Antiviral	4	9.213 × 10^−5^
Nevirapine	Anti-HIV	5	0.0246
Daclizumab	Anti-MS	4	0.0001
Natalizumab	Anti-MS	4	0.0001
Apomab	Anticancer	4	0.0048
Bevacizumab	Anticancer	4	0.0001
Cetuximab	Anticancer	4	0.0001
Ibritumomab	Anticancer	4	0.0001
temozolomide	Anticancer, brain	6	0.0414
Trastuzumab	Anticancer, breast	4	0.0002
Alemtuzumab	Anticancer, leukemia	4	0.0001
Dasatinib	Anticancer, leukemia	12	0.0018
Doxorubicin	Anticancer, leukemia	32	9.174 × 10^−7^
Gemtuzumab ozogamicin	Anticancer, leukemia	4	0.0001
Tositumomab	Anti-non-Hodgkins lymphoma	4	0.0001
Clodronate	Antiosteoporotic	5	0.0039
Raloxifene Hydrochloride	Antiosteoporotic	13	0.0305
Zoledronic acid	Antiosteoporotic	19	0.0198
Melatonin	Sleeping aid	11	5.279 × 10^−5^

## Data Availability

Data are contained within the article or Supplementary Material.

## Supplementary Materials

The following supporting information can be downloaded at: https://www.mdpi.com/article/10.3390/genes15080975/s1, Table S1: List of groups and sample sizes included in analyses, Table S2: Results of functional enrichment analysis using Toppfun, Table S3: Results of human disease phenotype analysis using Toppfun, Table S4: Results of drug-gene association analysis using Toppfun.
