# Supplementary material for: Simulated Microgravity Alters Gene Regulation Linked to Immunity and Cardiovascular Disease
Source: Genes (Basel). 2024 Jul 24;15(8):975. doi: 10.3390/genes15080975 (PMC11353732; doi:10.3390/genes15080975)
Supplement: Supplementary file 1 [file genes-15-00975-s001.zip › Suppl materials/Table S1.pdf]

Table S1. List of groups and sample sizes included in analyses. NL: Normally loaded control, HU: hindlimb unloading

Treatment and duration	Young female (3 months)	Young male (3 months)	Older male (9 months)
14 days NL	7	7	7
14 days HU	8	6	7
90 days NL	-	-	3
90 days HU	-	-	3
90 days NL + 90 days Recovery	-	-	4
90 days HU + 90 days Recovery	-	-	5

(-): Not included in study either due to insufficient sample size or unavailability of appropriately processed samples.
